# Quantitative reverse transcription PCR assay to detect a genetic marker of pyrethroid resistance in *Culex* mosquitoes

**DOI:** 10.1371/journal.pone.0252498

**Published:** 2022-08-08

**Authors:** Kelli M. Hager, Erick Gaona, Amy Kistler, Kalani Ratnasiri, Hanna Retallack, Miguel Barretto, Sarah S. Wheeler, Christopher M. Hoover, Eric J. Haas-Stapleton

**Affiliations:** 1 Alameda County Mosquito Abatement District, Hayward, CA, United States of America; 2 School of Public Health, University of California, Berkeley, Berkeley, CA, United States of America; 3 Chan Zuckerberg Biohub, San Francisco, CA, United States of America; 4 University of California, San Francisco, CA, United States of America; 5 Sacramento-Yolo County Mosquito and Vector Control District, Elk Grove, CA, United States of America; Fundacao Oswaldo Cruz Instituto Rene Rachou, BRAZIL

## Abstract

Pyrethroid insecticides are widely used to control mosquitoes that transmit pathogens such as West Nile virus (WNV) to people. Single nucleotide polymorphisms (SNP) in the knockdown resistance locus (*kdr*) of the *voltage gated sodium channel* (*Vgsc*) gene in *Culex* mosquitoes are associated with knockdown resistance to pyrethroids. RNAseq was used to sequence the coding region of *Vgsc* for *Culex tarsalis* Coquillett and *Culex erythrothorax* Dyar, two WNV vectors. The cDNA sequences were used to develop a quantitative reverse transcriptase PCR assay that detects the L1014F *kdr* mutation in the *Vgsc*. Because this locus is conserved, the assay was used successfully in six *Culex spp*. The resulting *Culex* RT*kdr* assay was validated using quantitative PCR and sequencing of PCR products. The accuracy of the *Culex* RT*kdr* assay was 99%. The L1014F *kdr* mutation associated with pyrethroid resistance was more common among *Cx*. *pipiens* than other *Culex* spp. and was more prevalent in mosquitoes collected near farmland. The *Culex* RT*kdr* assay takes advantage of the RNA that vector control agencies routinely isolate to assess arbovirus prevalence in mosquitoes. We anticipate that public health and vector control agencies may employ the *Culex* RT*kdr* assay to define the geographic distribution of the L1014F *kdr* mutation in *Culex* species and improve the monitoring of insecticide resistance that will ultimately contribute to effective control of *Culex* mosquitoes.

## Introduction

Many mosquitoes within the *Culex* genus that are present in California can transmit West Nile virus (WNV), St. Louis Encephalitis virus (SLEV), and filarial worms to humans and other animals [1]. WNV and SLEV are maintained in a bird-mosquito cycle by mosquitoes such as *Culex pipiens* Linneaus and *Culex erythrothorax* Dyar that preferentially feed on birds. *Culex tarsalis* Coquillett, another WNV vector, transition seasonally from ornithophilic to general feeders or when host availability is constrained [2, 3]. Humans and horses are considered dead-end hosts for these arboviruses because they generate low viremia, thereby preventing onward transmission [4, 5]. There have been over 7000 symptomatic human infections of WNV since it was introduced to California in 2003 [6, 7]. Vector control agencies interrupt disease transmission through environmental manipulation, biological or chemical control of adult and juvenile mosquitoes, and public education. Adulticides (pesticides that target adult mosquitoes), such as pyrethroids, are used to reduce mosquito abundance and pathogen transmission.

Pyrethroid insecticides preferentially bind to open voltage gated sodium channels (*Vgsc*) in neuronal membranes, preventing their closure. The open *Vgsc* leaves the membrane depolarized and the neuron unable to transmit signals among cells, resulting in paralysis (i.e., knockdown) and death of the insect [8, 9]. More than 50 knockdown resistance (*kdr*) mutations in the sodium channel gene are associated with pyrethroid resistance among arthropods [10]. The most common among *Culex* species is the L1014F single nucleotide polymorphism (SNP), which promotes closed state inactivation and knockdown resistance [11, 12].

Pyrethroids are commonly used to control structural and agricultural arthropod pests. The CDC considers mosquito populations resistant to an adulticide when knockdown or mortality rates are less than 90% in an adult mosquito bottle bioassay [13]. Increased use of pyrethroids in agricultural settings may contribute to pyrethroid resistance among a broad range of arthropods [14, 15]. Concerns with widespread pyrethroid resistance in mosquitoes prompted us to develop a quantitative reverse transcriptase-polymerase chain reaction (RT-qPCR) assay that detects the L1014F SNP in the *kdr* locus of *Culex* species. Our original goal was to develop this assay for use with *Cx*. *tarsalis*, but after comparing the cDNA sequences of other *Culex* vectors, we discovered the RT-qPCR assay produced a more conserved template compared to its DNA-based PCR counterparts [16–18].

Here we describe the development of a *Culex* RT*kdr* assay and application of the assay to map within Alameda County (California, USA) the frequency of the L1014F SNP that is associated with pyrethroid susceptibility (homozygous LL-1014) or resistance (homozygous FF-1014 and heterozygous LF-1014) [11]. The L1014S polymorphism in the *kdr* loci of the *vgsc* gene (heterozygous SF-1014) is associated with resistance to pyrethroid insecticides [19], and was detected in less than 5% of *Cx*. *pipiens* from the East Coast of the USA [16] but was not assessed by the *Culex* RT*kdr* assay because LF-1014 is reportedly more prevalent [5, 11].

## Methods

### 1. Mosquito collection

Adult mosquitoes from the environment were collected overnight from May—October of 2019 in Alameda County (California, USA) using encephalitis vector survey (EVS) traps (catalog number 2801A, BioQuip, Rancho Dominguez, CA) that were baited with dry ice [20]. The trapped mosquitoes were identified to species using a dissection microscope (Olympus SMZ800, Tokyo, Japan) and chill table (catalog number 1431, BioQuip, Rancho Dominguez, CA) [21]. A scientific collection permit was not required because the collections were made by a mosquito abatement district that was operating under the legislative authority of the California Health and Safety Code § 2040. Field studies did not involve endangered or protected species. The *Culex quinquefasciatus* Say strains (CqWV-1 and CqWV-2) and *Cx*. tarsalis strains (KNWR, (from the Kern National Wildlife Refuge [22, 23]) and Conaway (originally collected during 2019 in Woodland, California USA (GPS coordinates: 38.647287, -121.668173)) were maintained in an insectary prior to use [24]. Individual whole mosquitoes were placed into 2 ml microcentrifuge bead mill tubes that contained 2.8 mm ceramic beads (Fisher Scientific, Waltham, MA) and frozen at -20°C until use.

### 2. Nucleic acid extraction

Individual whole mosquitoes were homogenized in 200 μl of MagMAX Lysis/Binding Buffer that was diluted 1:2 in phosphate buffer saline for 45 s using a Fisherbrand Bead Mill 24 Homogenizer (Thermo Fisher Scientific, Waltham, MA). Nucleic acid was extracted using the MagMAX-96 Viral RNA Isolation Kit (which isolates both RNA and DNA) and the KingFisher Duo Prime Purification System programed with the MagMAX Pathogen Standard Volume software protocol as described by the manufacturer (Thermo Fisher Scientific, Waltham, MA) with the following exceptions: 80 μl of homogenate was extracted, magnetic beads were washed with 250 μl of wash solution, and the nucleic acid was eluted in 50 μl. Notably, we employed the same nucleic acid extraction method that is widely used by vector control agencies to test mosquitoes for the presence of arboviruses [25]. Alternatively, RNeasy Plus Mini Kits (Qiagen, Mississauga, Ontario, Canada) were used to extract nucleic acid from mosquitoes, as recommended by the manufacturer (Qiagen, Mississauga, Ontario, Canada). RNA and DNA concentration in the samples was measured using a NanoDrop 2000 Spectrophotometer (ThermoFisher Scientific, Waltham, MA), according to the manufacturer recommendations.

### 3. RNAseq of *Vgsc* gene

*Vgsc* sequences were recovered from the host fraction of a metatranscriptomic RNAseq dataset derived from total RNA extracted from *Cx*. *erythrothorax* (N = 44) and *Cx*. *tarsalis* (N = 26) single mosquitoes collected from across California using EVS traps [26]. Sample collection, total RNA extraction, and paired-end mNGS RNAseq from each of the single mosquito specimens that served as input data here are described elsewhere ([26]; sequence related archive: https://www.ncbi.nlm.nih.gov/sra/PRJNA605178). Raw fastq R1 and R2 data from each mosquito RNAseq dataset were first compressed to a unique set of reads sharing < 95% sequence identity via CD-HIT software [27, 28]. Translated blastx alignment of the resulting R1 and R2 reads with a representative *Vgsc* protein sequence from *Cx*. *quinquefasciatus* (NCBI protein accession AFW98419.1; [29] was applied to identify deduplicated R1 and R2 reads from each mosquito sample which showed >50% of their length aligned with >90% identity to the *Cx*. *quinquefasciatus Vgsc* reference sequence. Seqtk software (https://github.com/lh3/seqtk) was used to compile the separate *Cx*. *erythrothorax* and *Cx*. *tarsalis* fastq reads that met these criteria from the 44 *Cx*. *erythrothorax* or 26 *Cx*. *tarsalis* individually deduplicated datasets. Partners of unpaired reads included in each pool were identified and included to ensure a full complement of paired reads, including additional potentially divergent *Vgsc* sequences that were not captured in the alignment step.

A total of 410 *Cx*. *tarsalis* input read pairs and 481 *Cx*. *erythrothorax* read pairs were carried forward from this step. Trimmomatic software [30] was used to remove the sequencing library adapter sequences, along with low quality terminal bases of the reads. The resulting paired-end pooled datasets were each then separately used as input for SPAdes [31] paired-end *de novo* assembly of *Vgsc* transcripts. To facilitate *Vgsc* contig coverage analysis, read pools were aligned back to each of the identified *Vgsc* contigs via Bowtie2 [32].

The *Cx*. *tarsalis* and *Cx*. *erythrothorax* contig assemblies were aligned to the NCBI nt and nr databases via blastn and blastx, respectively, to identify the set of *de novo* assembled contigs that corresponded to *Vgsc* transcripts. The most closely related sequences in NCBI to this contig corresponded to several *Culex* complete *Vgsc* nucleotide and protein coding sequences. The best match was the *Cx*. *pipiens pallens* strain SS sodium channel mRNA (NCBI accession numbers KY171978.1 and ARO72116.1), showing >95% overall sequence identity at both the nucleotide and amino acid level. The *Cx*. *erythrothorax* contigs were not joined in the initial *de novo* assembly; however, the blastn and blastx alignment termini indicated a short (< 10 bp) region of overlapping sequence at the ends of these 2 contigs. Manual joining of these 2 contigs generated a 6709 bp contig that encodes an uninterrupted open reading frame of 2109 amino acids, and additional flanking 283 bp of 5’utr and 99 bp of 3’utr sequences. The best match was the *Cx*. *quinquefasciatus* isolate S-Lab sodium channel mRNA, complete cds (NCBI accession numbers EU817515.1 and ARO72116.1), showing >95% overall sequence identity at both the nucleotide and amino acid level. The Genbank accession numbers for the recovered *Cx*. *erythrothorax* and *Cx*. *tarsalis Vgsc* transcript sequences are MW176091 and MW176090, respectively. Resulting assemblies were manually reviewed via Geneious software (version 2019.0.4; https://www.geneious.com/) to generate final contig consensus sequences.

### 4. Assessing the *kdr* SNP genotype using the *Culex* RT*kdr* assay

**Fig 1:**
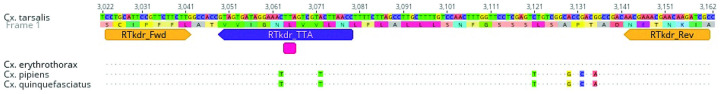
*Culex* RT*kdr* assay design and alignments. Sequence alignment of *Cx*. *tarsalis* (MW176090), *Cx*. *erythrothorax* (MW176091), *Cx*. *pipiens* (KY171978) and *Cx*. *quinquefasciatus* (EU817517) with *Cx*. *tarsalis* (MW176090) as the reference sequence at the *kdr* locus. RT*kdr* primers and probe are depicted in orange and purple, respectively. Disagreements to the consensus sequence are highlighted while agreements are noted as a dot.

The primer and probe sequences to detect the *kdr* SNP were designed using Primer3Plus software (Table 1; [33]) with the cDNA sequences of *Vgsc* from *Cx*. *tarsalis* and *Cx*. *erythrothorax* (GenBank No. MW176090 and MW176091, respectively). The probe that detected the L-1014 SNP (RTkdr_TTA) was labeled with fluorescein (FAM) and the probe that detected the F-1014 SNP (RTkdr_TTT) was labeled with hexachlorofluorescein (HEX; Integrated DNA Technologies, Coralville, Iowa). Geneious Prime software (Geneious Prime 2022.0.1; https://www.geneious.com/) was used to align *Cx*. *tarsalis (*MW176090), *Cx*. *erythrothorax (*MW176091), *Cx*. *pipiens* (KY171978), and *Cx*. *quinquefasciatus* (EU817517) with *Cx*. *tarsalis* as the reference sequence. BLAST was used to determine the percent identity of each species to the *Cx*. *tarsalis* amplicon sequence. Primers and probes used for the *Culex* RT*kdr* assay and subsequent sequencing are indicated on the alignment (Fig 1).

**Table 1:** Primers and probes. Underlined text indicates the location of the 1014 codon.

Name	Sequence (5’ → 3’)
**Primers**	
RTSeq_Fwd	ATCTGACGTTTGTGCTCTGC
RT*kdr*_Fwd	CCTGCATTCCGTTCTTCTTG
RT*kdr*_Rev	GCGATCTTGTTCGTTTCGTT
**Probes**	
RT*kdr*_TTA	FAM-GGTTAAGTA/ZEN/CGACTAAGTTTCCTATCACTAC-3IABkFQ
RT*kdr*_TTT	HEX-GGTTAAGTA/ZEN/CGACAAAGTTTCCTATCACTAC-3IABkFQ

For *Culex* RT*kdr* genotyping, the Taqman Fast Virus 1-Step Master Mix (Thermo Fisher Scientific, Waltham, MA) was prepared as described by the manufacturer using 1 μl of template RNA (48.8–144.8 ng/μl), primers diluted to 900 nM and probes diluted to 250 nM. PCR plates were vortexed for 10 s at the highest setting, centrifuged for 15 s (MPS 1000 Mini PCR Plate Spinner, Labnet International, Inc., Edison, NJ) and subsequently analyzed with a QuantStudio 5 Real-Time PCR System (Thermo Fisher Scientific, Waltham, MA) using the Genotyping setting. RT-qPCR cycling conditions were as follows: 50°C for 5 min, 95°C for 20 s, followed by 40 cycles of 95°C for 3 s and 60°C for 30 s. Primer and probe concentration and PCR cycling conditions were optimized to discriminate homozygous and heterozygous genotypes. Allele controls were added in the form of a no template control and a known susceptible control. A known resistant control was also included for each of the former except *Cx*. *erythrothorax* because a resistant specimen of that species was not found in the current study. Amplification curves were reviewed manually to ensure algorithm accuracy. We defined ΔCT (Eq 1) as the cycle threshold (CT) of the mutant (RT*kdr*_TTT) probe–CT wildtype (RT*kdr*_TTA) probe [34]. If a probe did not amplify, a CT value of 40 (final cycle number) was used to calculate ΔCT values. Amplification curve characteristics were used provisionally to determine the *kdr* genotype by plotting the fluorescence emission intensity of the reporter dye subtracted by the baseline signal (ΔRn) against cycle number. K-means cluster analysis was used subsequently to categorize *kdr* genotype by ΔCT values by setting the number of clusters to 3 (one for each potential *kdr* genotype) and fewer than 10 iterations to evaluate changes in clustering centers using IBM SPSS Statistics software (software version 25; IBM, Armonk, NY USA).


$$\Delta CT=CT_{RTkdr\_TTT}-CT_{RTkdr\_TTA}$$
Eq 1:

### 5. Insecticide susceptibility assays

CDC bottle bioassays were conducted to evaluate the resistance of adult female mosquitoes to insecticides, according to CDC guidelines [13]. Three replicate bottles were evenly coated with 1 ml of technical grade insecticide (43 μg permethrin or 22 μg deltamethrin) that was diluted in acetone. Control bottles contained only acetone diluent. The diluent was evaporated from the bottles in the dark at room temperature. Adult female mosquitoes were transferred to the bottles (21–23 mosquitoes per bottle), and the number of knocked down mosquitoes was recorded at 15 min intervals for 120 min. A mosquito was recorded as knocked down if it could not stand unaided when the bottle was gently rotated; otherwise, the mosquito was counted as not knocked down. Mosquitoes from one replicate bottle of the Conaway strain were separated as knocked down or not, tested with the *Culex* RT*kdr* assay, and the RT-PCR products sequenced (described below). Resistance ratios were calculated using the proportion of knocked down mosquitoes at the 45 min time point when average knockdown was less than 100% with those from the Conaway strain in the denominator. Fisher’s Exact Test was used to evaluate associations between *kdr* genotype and knockdown status (*i*.*e*., whether the mosquitoes were knockdown or not) using IBM SPSS Statistics software (software version 25; IBM, Armonk, NY USA).

### 6. Assessing the *Culex* RT*kdr* assay using *Cx*. *pipiens* quantitative PCR (qPCR) Taqman assay

The *Cx*. *pipiens* quantitative PCR (qPCR) Taqman assay that was developed previously and used genomic DNA as the template [17] was utilized to assess the *Culex* RT*kdr* assay using *Cx*. *pipiens* individuals that were collected with EVS traps in the field (N = 75). The protocol for the Taqman Multiplex Master Mix (ThermoFisher Scientific, Waltham, MA) was followed with the following modifications: BSA was excluded and nucleic acid that was isolated using the MagMAX-96 Viral RNA Isolation Kit (described above) was used as the template. Discordant samples were evaluated by Sanger sequencing the PCR products.

### 7. Sanger sequencing of RT-PCR products

A primer upstream of the L1014 SNP in the *Cx*. *tarsalis* cDNA sequence (RTseq_Fwd) was used with the RT*kdr*_Fwd primer to produce a 373 bp RT-PCR product for sequencing (Table 1, Fig 1). The RT*kdr*_Fwd primer was too near to the L1014F SNP to be used for sequencing. Primer, probe and template concentrations and RT-PCR cycling conditions to generate PCR products for sequencing were as described above. RT-PCR products were submitted to Elim Biopharmaceuticals (Hayward, CA) for PCR cleanup and Sanger sequencing. Sequences were aligned to the *Cx*. *tarsalis Vgsc* mRNA sequence using MUSCLE [35] to locate the *kdr* SNP. Chromatograms were examined using 4Peaks software (Nucleobytes, Amsterdam, The Netherlands).

### 8. Assessing the geographic distribution of the *kdr* SNP

Tableau Software (Seattle, WA) was used to map the geographic distribution of the L1014F *kdr* mutation in mosquitoes that were collected in Alameda County (CA, USA) using EVS traps. The base map used in Tableau Software was the Topo Base Map from the United States Geological Survey [36]. Allelic data for mosquitoes that were collected within 1 km of each other were combined. The trap sites were binned into two geographic regions, bayside and inland, that are separated by the San Francisco East Bay Hills, a natural boundary that limits movement of mosquitoes between the two regions. The distribution of alleles that are associated with susceptibility or resistance to pyrethroid insecticides (LL-1014, LF-1014, and FF-1014) was assessed by mosquito species and by geographic region (inland and coastal) within Alameda County. The resistance allele frequency (F_(FF,LF)_) in each population was estimated using Eq 2 where N_FF_ was the number of FF-1014 mosquitoes, N_LF_ was the number of LF-1014 mosquitoes, and N the mosquito population size.


$$F_{\left(FF,LF\right)}=(2N_{FF}+N_{LF})/2N$$
Eq 2. Equation for calculating frequency of the *kdr* SNPs that are associated with resistance to pyrethroid insecticides.

Associations between genotype (Y; LL-1014, LF-1014, or FF-1014) mosquito species (Species; *Cx*. *tarsalis*, or *Cx*. *pipiens*), region of collection (Region; bayside or coastal), and land use surrounding the collection site (LandUse; wildlife, urban, industrial, or agriculture) were estimated using an ordinal logistic regression model with the ordered outcome categories of LL-1014, LF-1014, and FF-1014 [37, 38]. *Culex erythrothorax* was excluded from models because no resistant alleles (LF-1014 or FF-1014) were observed in this study for that species. Models were fit using the polr function from the MASS [39] package in R Software (version 3.5.0; [40]) and used to estimate unadjusted and adjusted odds ratios (OR) for each variable. Adjusted odds ratios were derived from a saturated model that included all covariates at once (Eq 3) whereas unadjusted odds ratios were derived from models with only the covariate of interest included (S1 File includes the R Software code that was used for the models). Confidence intervals that did not cross the null (OR = 1) indicated that the association was significant. P-values were estimated by comparing t-values from each regression model to a standard normal distribution (S1 File). Figures were generated using Prism (GraphPad Software, San Diego, CA) or ggplot2 software [41].


$$logit\;(P(Y\leq j))=\beta_{0j}–\beta_{1}Species–\beta_{2}Region–\beta_{3}LandUse$$
Eq 3. Ordinal logistic regression saturated model

## Results & discussion

### 1. Sequence alignments

**Fig 2:**
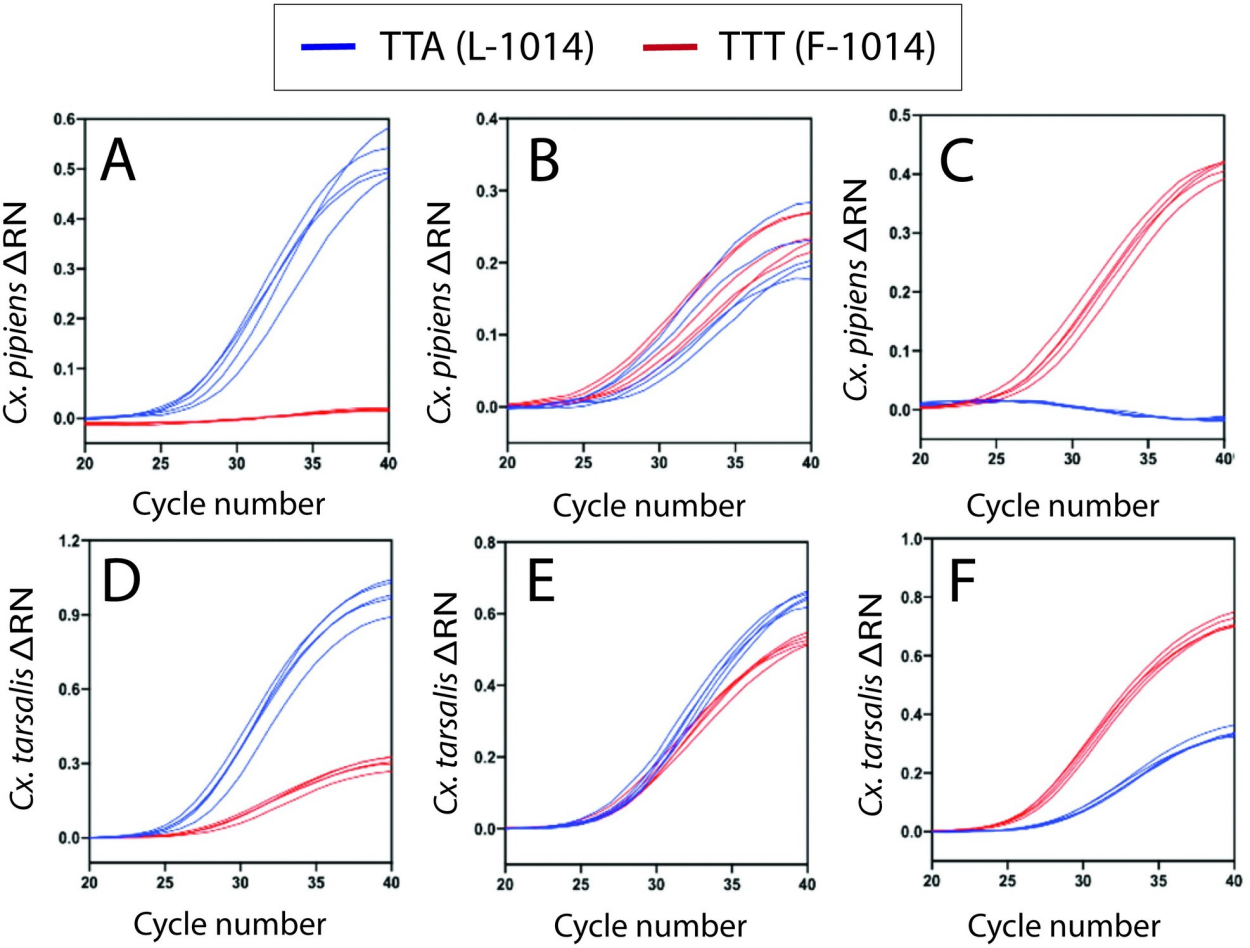
Amplification plots of the *Culex* RT*kdr* assay for adult *Cx*. *pipiens* and *Cx*. *tarsalis* captured in Alameda County using encephalitis vector survey traps. Amplification plots (ΔRN vs Cycle Number) with the RT*kdr*_TTA probe labeled in blue and RT*kdr*_TTT probe in red. Genotype assignments based upon whether one or both probes amplified in a *Culex* RT*kdr* assay, and subsequently confirmed using ΔCT values (see Fig 3). **(A)** *Culex pipiens* homozygous LL-1014 **(B)** *Culex pipiens* heterozygous LF-1014 **(C)** *Culex pipiens* homozygous FF-1014 (**D)** *Culex tarsalis* homozygous LL-1014 **(E)** *Culex tarsalis* heterozygous LF-1014 **(F)** *Culex tarsalis* homozygous FF-1014.

RNAseq of the mosquito fraction from the metagenome recovered a single 6878 bp *Cx*. *tarsalis* contig and two 6364 bp and 506 bp *Cx*. *erythrothorax* contigs that contained *Vgsc* sequences. The *Cx*. *tarsalis* 6878bp contig encompassed an uninterrupted 2113 amino acid open reading frame, with additional 5’ 321 bp and 3’ 218 bp flanking terminal sequences. The *Vgsc* -1 cDNA sequences for *Cx*. *erythrothorax* (GenBank No. MW176091), *Cx*. *pipiens* (GenBank No. KY171978) and *Cx*. *quinquefasciatus* (GenBank No. EU817517) were aligned to *Cx*. *tarsalis* (GenBank No. MW176090) using blastn. There was 96% identity for *Cx*. *pipiens* and *Cx*. *quinquefasciatus* while *Cx*. *erythrothorax* had 100% identity to *Cx*. *tarsalis* across the amplicon region. The forward and reverse primers matched 100% for all three species. There were two mismatched nucleotides between the probes and template for *Cx*. *pipiens* and *Cx*. *quinquefasciatus* in the *Culex* RT*kdr* assay (Fig 1). However, the mismatches did not prevent the probes from interacting with the template to produce amplification curves and ΔRN values in the *Culex* RT*kdr* assay (Fig 2, S1 Fig).

### 2. Interpreting *Culex* RT*kdr* assay results

**Fig 3:**
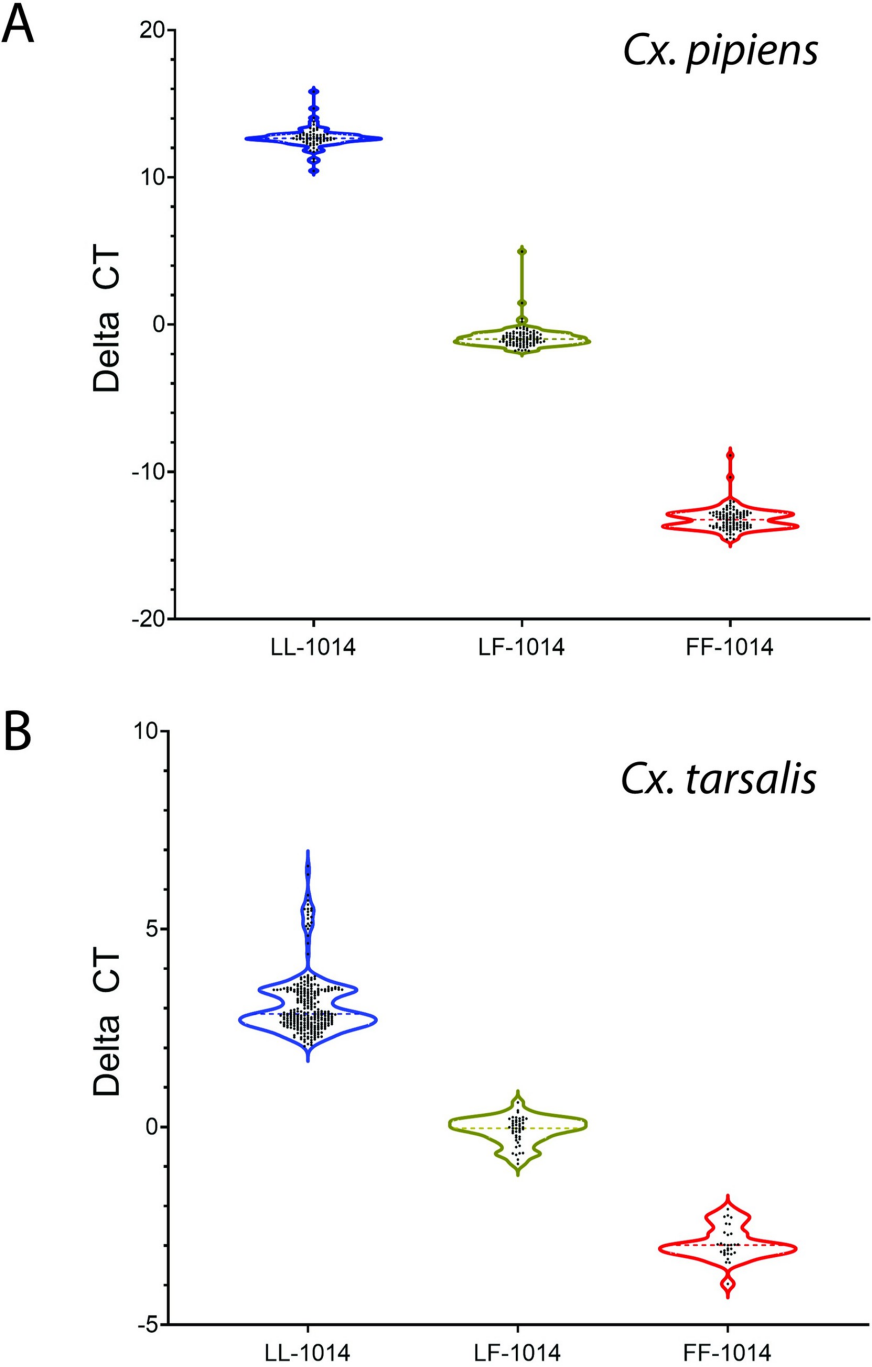
Genotype of *Cx*. *pipiens* and *Cx*. *tarsalis* at *kdr* locus. Violin plots showing ΔCT values by *kdr* genotype for *Cx*. *pipiens* (A) and *Cx*. *tarsalis* (B). Ellipses indicate ΔCT values and dash lines within each violin plot indicate median ΔCT values. Red indicates homozygous (FF-1014) yellow indicate heterozygous (LF-1014), and blue indicate homozygous (LL-1014) genotypes.

The *kdr* genotypes were provisionally assigned for *Cx*. *pipiens* and *Cx*. *tarsalis* based upon whether there was a substantial increase in the fluorescence of FAM, HEX, or both (Fig 2), as was done previously for a quantitative PCR assay that used genomic DNA to assess *kdr* genotypes in *Cx*. *pipiens pallens* [17]. For the *Culex* RT*kdr* assay, increased FAM fluorescence indicated a homozygous LL-1014 genotype (Fig 2A and 2D) and substantially increased HEX fluorescence indicated a homozygous FF-1014 genotype (Fig 2D and 2F). A similar quantity of FAM and HEX fluorescence indicated that the specimen had a heterozygous LF-1014 genotype (Fig 2B and 2E). The ΔCT values that were generated using the *Culex* RT*kdr* assay used to assign a *kdr* genotype to individual mosquitoes. To do so, ΔCT values from the *Culex* RT*kdr* assay were analyzed using k-means cluster analysis to determine cluster centers for each *kdr* genotype category (genotype FF-, LF-, or LL-1014; N = 264 and 360 ΔCT values for *Cx*. *pipiens* and *Cx*. *tarsalis*, respectively). Convergence of the change in k-means cluster centers for ΔCT values was achieved in 7 iterations for each species. The final cluster centers (*i*.*e*., median ΔCT values) for *Cx*. *pipiens* were -13.212, -0.871, and 12.691; while for *Cx*. *tarsalis* they were -2.944, -0.089, and 3.107. For *Cx*. *pipiens*, ΔCT values greater than 5.0 were categorized as LL-1014, ΔCT values less than -4.0 were LF-1014, and ΔCT values between 4.9 and -2.0 were LF-1014 (Fig 3A). For *Cx*. *tarsalis*, ΔCT values greater than 2.0 were categorized as LL-1014, ΔCT values less than -2.0 were LF-1014, and ΔCT values between 1.0 and -1.0 were LF-1014 (Fig 3B). The ΔCT values had a significant impact on assigning the *kdr* genotype in the k-means cluster analysis (ANOVA, *Cx*. *pipiens*: *F*(2, 261) = 23644.6, p < 0.001; *Cx*. *tarsalis*: *F*(2, 357) = 1228.5, p < 0.001). ΔCT values for *Cx*. *erythrothorax* resembled those for the LL-1014 genotype of *Cx*. *tarsalis*, and was the only genotype detected for that species.

Atypical amplification curves were occasionally observed for *Cx*. *pipiens* samples (<5% of total), suggesting these mosquitoes may have been misidentified and were instead *Cx*. *erythrothorax*. *Culex pipiens* and *Cx*. *erythrothorax* are morphologically similar and can be mistaken for each other [4]. To determine if the *Cx*. *pipiens* with uncharacteristic amplification curves may have been misidentified, we tested them using the *Cx*. *pipiens* qPCR assay that only produces a PCR product with DNA isolated from *Cx*. *pipiens* or *Cx*. *quinquefasciatus* [17]. Each of those samples failed to amplify a product with the *Cx*. *pipiens* qPCR assay, providing additional evidence that the mosquitoes may have indeed been *Cx*. *erythrothorax*.

*Culex tarsalis*, *Cx*. *pipiens*, *and Cx*. *erythrothorax* were the most prevalent *Culex* species collected during the study period. We also tested *Cx*. *quinquefasciatus* (strains CqWV-1 and CqWV-2), *Culex stigmatosoma* Dyar, and *Culex apicalis* Adams. The low sample size for these species did not allow us to determine ΔCT value ranges for assigning a *kdr* genotype as was done for *Cx*. *pipiens* and *Cx*. *tarsalis* (Fig 3). However, the amplification curves (S1 Fig) suggest that the *Culex* RT*kdr* assay may be effective for those species as well, but additional validation would be needed to confirm. Amplification curves from the *Culex* RT*kdr* assay for *Cx*. *quinquefasciatus* demonstrate that individuals of the strain CqWV-1 had the LL-1014 *kdr* genotype and may be susceptible to pyrethroids, while CqWV-2 had the FF-1014 genotype and may be relatively resistant to pyrethroids. Bottle bioassays would need to be conducted with both strains to determine whether the *kdr* genotype confers functional resistance to pyrethroids.

### 3. Assessing assay efficacy

#### 3.1 Insecticide susceptibility assays

**Fig 4:**
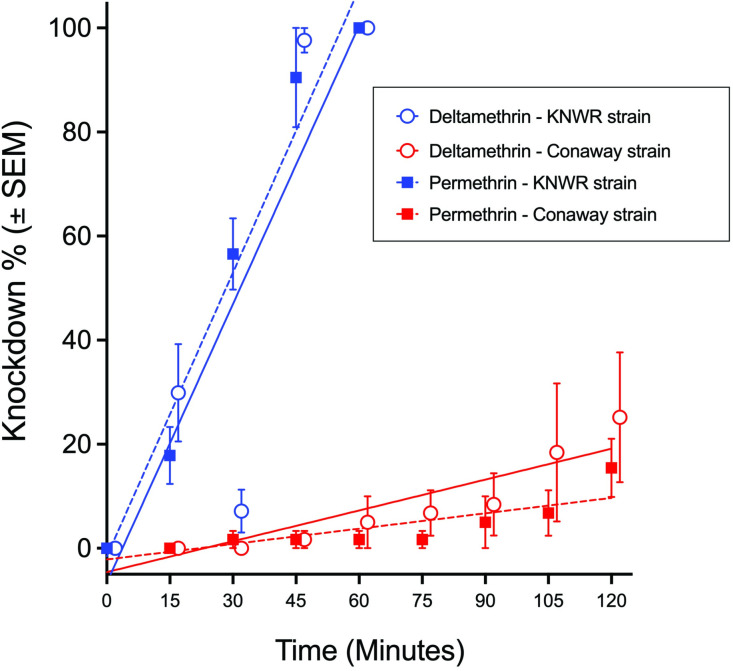
Bottle bioassay of *Cx*. *tarsalis* strains KNWR and Conaway with permethrin or deltamethrin. Closed squares indicate bottle bioassays using permethrin and open circles indicate deltamethrin bottle bioassays. Blue symbols and regression lines denote bottle bioassays with KNWR strain *Cx*. *tarsalis*, and red symbols and regression lines indicate bottle bioassays with Conaway strain *Cx*. *tarsalis*. Deltamethrin symbols are offset by 2 min for clarity. Equation of lines: Deltamethrin: susceptible KNWR strain, Y = 1.785–X—6.627 (R^2^ = 0.7403); resistant Conaway strain, Y = 0.1977*X– 4.588 (R^2^ = 0.3585); Permethrin: susceptible KNWR strain, Y = 1.818–X—1.553 (R^2^ = 0.9283); resistant Conaway strain, Y = 0.0870*X– 2.156 (R^2^ = 0.3584).

Two laboratory strains of *Cx*. *tarsalis* (KNWR and Conaway) were assessed for susceptibility to permethrin or deltamethrin using CDC bottle bioassays. Both insecticides are routinely used by vector control agencies to reduce the abundance of adult *Culex spp*. mosquitoes, and resistance to these insecticides was present in mosquitoes that were collected from the field [19, 24, 42]. Knockdown in the CDC bottle bioassay was on average less than 5% for mosquitoes placed in bottles that contained only diluent. At the 60 min time point, all KNWR strain mosquitoes displayed the knockdown behavior in response to permethrin and deltamethrin (Fig 4). At the 45 min time point, when the average knockdown was less than 100% for all treatments, the Conaway strain was 54.5- and 58.8-fold more resistant to permethrin and deltamethrin, respectively. Resistance ratios of these magnitudes indicate that the Conaway strain was highly resistant to the insecticides. At 120 min, 15 ± 5% of the Conaway strain mosquitoes were knocked down in response to permethrin and 25 ± 10% to deltamethrin (Fig 4). The US Centers for Disease Control and Prevention classifies a population as potentially resistant when knockdown or mortality in a CDC bottle bioassay is below 90% at two hours (120 min) after exposure to the insecticide [43, 44]. Deltamethrin and permethrin were equally effective in knocking down the KNWR strain mosquitoes (Fig 4, blue regression lines; ANCOVA, F (1,26) = 0.001, p = 0.9212). The regression line slopes for the Conaway strain mosquitoes exposed to permethrin or deltamethrin were similar as well, pointing to the insecticides also having similar efficacy in the Conaway strain mosquitoes (Fig 4, red regression lines; ANCOVA, F (1,50) = 2.804, p = 0.1003). However, the slope of the regression lines were significantly different for the Conaway and KNWR strain mosquitoes that were exposed to permethrin or deltamethrin (Fig 4; permethrin: square symbols; ANCOVA; F (1,38) = 267.6, p < 0.0001; deltamethrin: elliptical symbols; F (1,38) = 53.37, p < 0.0001), suggesting that the Conaway strain mosquitoes were more resistant to both pyrethroid insecticides (Fig 4, Conaway: red regression lines; KNWR: blue regression lines). Cross-resistance to permethrin and deltamethrin occurs in field-collected populations of *Cx*. *quinquefasciatus*, although deltamethrin is often slightly more effective than permethrin at knocking down mosquitoes [45, 46]. Although not evaluated herein, synergists can be included with pyrethroids to inhibit cytochrome P450 monooxygenases (CYP) and increase knockdown in mosquitoes [47]. However, when multiple insecticide resistance pathways are present, such as increased CYP expression and mutant *kdr* genotypes, adding synergists to pyrethroids may not be sufficient for controlling highly resistant mosquitoes [48].

**Fig 5:**
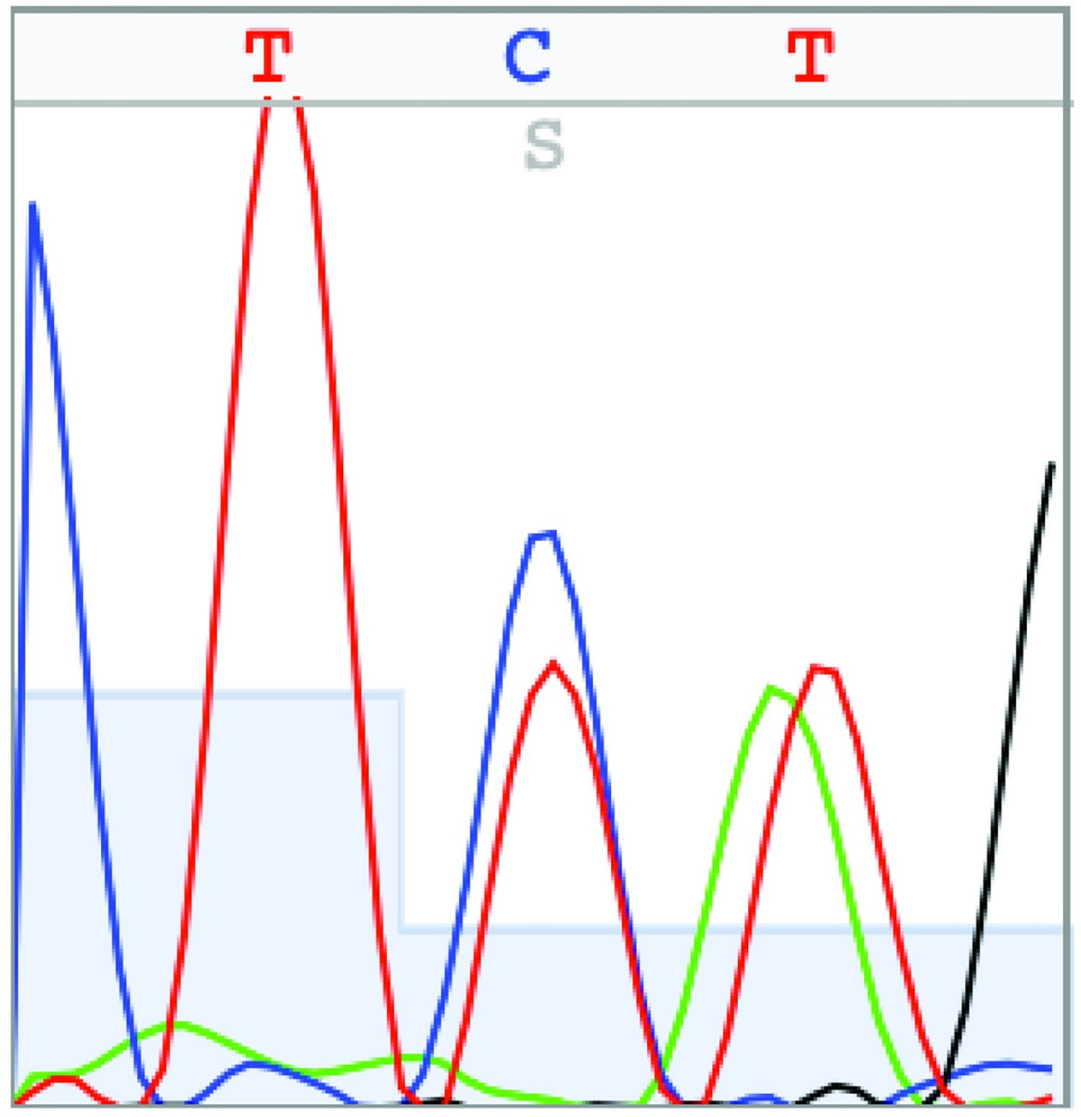
Chromatogram showing the SF-1014 (i.e., L1014S) heterozygote in the Conaway strain mosquitoes.

The *kdr* genotype of the Conaway strain mosquitoes from the permethrin and deltamethrin bottle bioassays was determined using the *Culex* RT*kdr* assay. The results of the *Culex* RT*kdr* assay were compared to those obtained by Sanger sequencing the RT-PCR products that were generated using the RTSeq_Fwd and RTSeq*kdr*_Rev primers. All of the mosquitoes that were not knocked down after exposure to permethrin or deltamethrin were FF-1014 in the *Culex* RTkdr assay (Table 2). Of those mosquitoes that were knocked down, 60% and 57% of those that were exposed to permethrin or deltamethrin, respectively, had the FF-1014 genotype, with the remainder having a genotype that is associated with resistance to pyrethroids. The *Culex* RT*kdr* assay and Sanger sequencing results agreed for each sample except for six individuals that were indicated as LF-1014 by the *Culex* RT*kdr* assay, but sequencing showed them to be SF-1014 (Table 2, Fig 5). The LF-1014 genotype is associated with resistance to pyrethroids [11] while the SF-1014 genotype, which is not detected by the *Culex* RT*kdr* assay, is associated with cross-resistance between pyrethroids and DDT [16]. DDT was banned by the US Environmental Protection Agency during 1972, but persists in the environment [49] and may have exerted a selective pressure on mosquitoes in the Conaway rice field that contributed to propagating the SF-1014 genotype.

**Table 2:** Genotype of the Conaway strain mosquitoes from the bottle bioassays. Percentage values are for each knockdown state (No or Yes) in the bottle bioassay, number of mosquitoes are in parenthesis and superscripts indicate if the genotype was determined with the *Culex* RT*kdr* Assay and confirmed with Sanger sequencing^a^, or only Sanger sequencing^b^.

		Genotype from *Culex* RT*kdr* Assay^a^ or Sequencing^b^			
Insecticide in assay	Knockdown after 120 min	LL-1014^a^	LF-1014^a^	FF-1014^a^	SF-1014^b^
Permethrin	No	0% (0)	0% (0)	100% (11)	0% (0)
	Yes	7% (1)	13% (2)	60% (9)	20% (3)
Deltamethrin	No	0% (0)	0% (0)	100% (12)	0% (0)
	Yes	0% (0)	27% (4)	57% (8)	20% (3)

Fisher’s exact test (2-sided) was used to determine if there was a significant association between *kdr* genotype and knockdown status for Conaway strain mosquitoes that were exposed to permethrin or deltamethrin in the CDC bottle bioassay (Table 2). For mosquitoes exposed to deltamethrin, there was a significant association between the two variables (p = 0.005), while there was not a significant association for mosquitoes exposed to permethrin (p = 0.138). The small sample size and lack of all genotypes in the two bioassay outcomes (knockdown or not) limits the conclusions that can be drawn. However, we demonstrate that determining the *kdr* genotype of mosquitoes from a bottle bioassay is feasible using the *Culex* RT*kdr* assay. These limited results suggest that there was an association between *kdr* genotype and knockdown status for Conaway strain mosquitoes exposed to deltamethrin in a CDC bottle bioassay with the FF-1014 *kdr* genotype predominating in individuals that were knocked down. Vector control agencies can quickly detect arbovirus-infected mosquitoes if they employ rapid antigen-based tests or quantitative PCR, obtaining test results in minutes to hours after the mosquitoes are collected. The susceptibility of mosquitoes to an insecticide must be considered if the insecticide application is to be efficacious, and public health protected [50]. The CDC bottle bioassay is considered by the CDC to be an effective test for assessing the susceptibility of a field-collected population of mosquito to an insecticide [50]. However, conducting CDC bottle bioassays is relatively labor-intensive, and may not be feasible in the time frame needed to engage mosquito control efforts in a timely manner, particularly if sufficient quantities of adult female mosquitoes cannot be collected from where the arbovirus-infected mosquitoes are discovered. Molecular tests of insecticide susceptibility, such as the *Culex* RT*kdr* assay, provide rapid results that may indicate whether a population of mosquitoes is likely to be resistant to a pyrethroid insecticide, thereby serving as a potential proxy for the CDC bottle bioassay.

#### 3.2 Comparison of *Culex* RT*kdr* assay with *Cx*. *pipiens* qPCR Taqman assay

To determine the fidelity of the *Culex* RT*kdr* assay, individual *Cx*. *pipiens* mosquitoes that were collected using EVS traps were evaluated with the *Culex* RT*kdr* assay using an RNA template and the *Cx*. *pipiens* qPCR assay using a genomic DNA template. Three specimens (4%) that failed to amplify a product after 30 PCR cycles in the *Culex* RT*kdr* assay were excluded. Of the remaining mosquitoes, 69/72 (96%) were concordant across both assays (Table 3). Discordant results were sequenced to determine the correct *kdr* genotype. Sanger sequencing chromatograms for the three (4%) discordant samples indicated the mosquitoes were heterozygous and in agreement with the *Culex* RT*kdr* results, demonstrating that the *Culex* RT*kdr* assay was highly accurate.

**Table 3:** Assessing the *Culex* RT*kdr* assay using a *Cx*. *pipiens* qPCR Taqman assay.

Assay	*Culex* RT*kdr*			*Cx*. *pipiens* qPCR		
SNP Genotype	LL-1014	LF-1014	FF-1014	LL-1014	LF-1014	FF-1014
N	23	26	23	23	23	26

#### 3.3 Comparing the *Culex* RT*kdr* assay with Sanger sequencing

The *kdr* genotype determinations that were made using the *Culex* RT*kdr* assay were compared to those obtained by Sanger sequencing RT-PCR products that were generated using RTSeq_Fwd RT*kdr*_Rev primers. Across five *Culex* species that were collected using EVS traps (*Cx*. *pipiens*, *Cx*. *tarsalis*, *Cx*. *erythrothorax*, *Cx*. *stigmatosoma*, and *Cx*. *apicalis*) greater than 99% of the field-collected specimens were concordant between the Sanger sequencing and *Culex* RT*kdr* assay results (N = 190; Table 4). The single discordant sample was misidentified as FF-1014 by the *Culex* RT*kdr* assay, but the chromatogram revealed two peaks at the SNP location, indicating the mosquito was LF-1014. Two strains of *Cx*. *quinquefasciatus* were assessed similarly, with the results from the *Culex* RT*kdr* assay and Sanger sequencing agreeing for all individuals that were assesses (Table 4). Using the sequencing results as the correct result, we found the accuracy of the *Culex* RT*kdr* assay was greater than 99%. High accuracy is common among both qPCR and RT-qPCR assays [51, 52].

**Table 4:** Validating the *Culex* RT*kdr* assay by Sanger sequencing RT-PCR products. Homozygous LL-1014 (LL), heterozygous LF-1014 (LF), homozygous FF-1014 (FF).

Species (N)	*Culex* RT*kdr* Assay			Sanger Sequencing		
	LL	LF	FF	LL	LF	FF
*Cx*. *pipiens* (51)	17	13	21	17	13	21
Cx. tarsalis (97)	18	17	62	18	18	61
*Cx*. *erythrothorax* (16)	16	0	0	16	0	0
*Cx*. *quinquefasciatus* CqWV-1 (10)	10	0	0	10	0	0
*Cx*. *quinquefasciatus* CqWV-2 (10)	0	0	10	0	0	10
*Cx*. *stigmatosoma* (5)	5	0	0	5	0	0
*Cx*. *apicalis* (1)	0	0	1	0	0	1
Total (190)	66	30	94	66	31	93

### 5. Geographic distribution of a *kdr* allele: Case study

**Fig 6:**
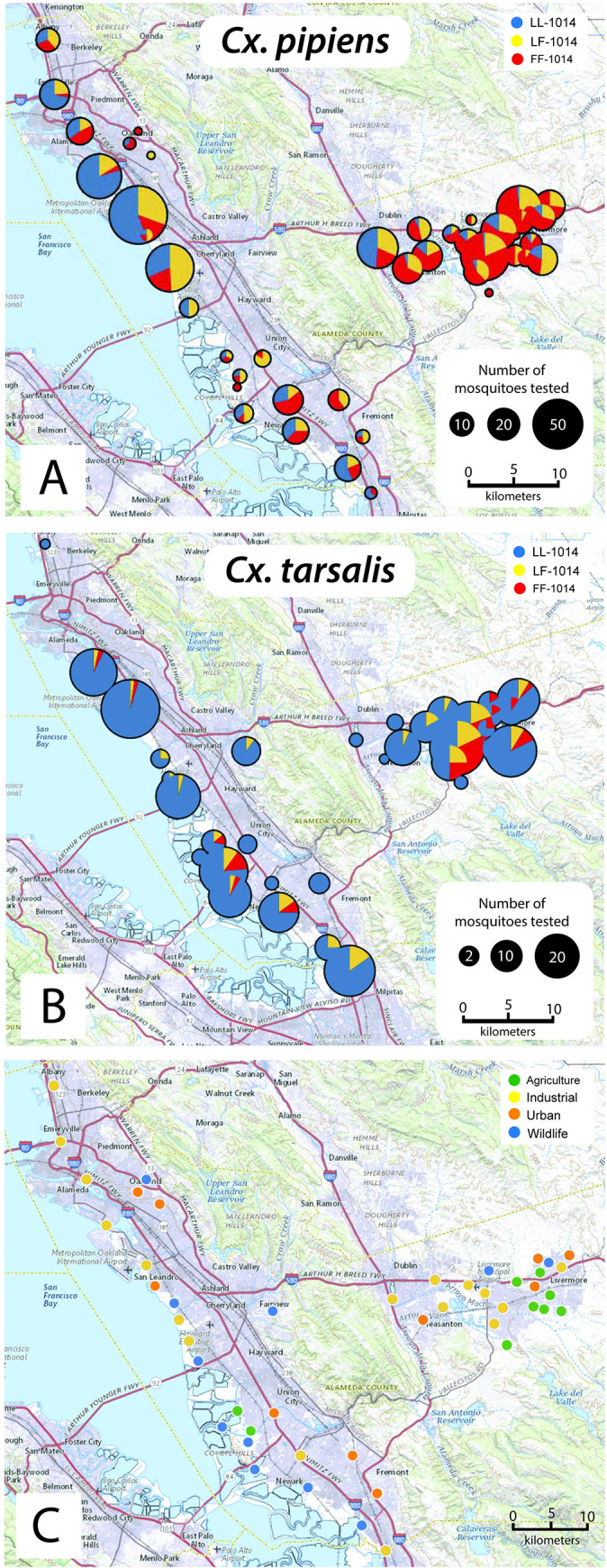
Geographic distribution of the *kdr* genotypes for **(A)** *Cx*. *pipiens* and **(B)** *Cx*. *tarsalis*, and **(C)** land use types in Alameda County. **(A, B)** The size of each pie chart indicates the relative number of mosquitoes that were assessed for the *kdr* genotype at each site. The *kdr* genotypes are color coded as: blue for LL-1014, yellow for LF-1014 and red for FF-1014. **(C)** Locations of land use types in Alameda County where EVS traps were placed to collect mosquitoes (green ellipse indicate an agricultural site, yellow an industrial site, orange an urban site and blue a wildlife site).

**Fig 7:**
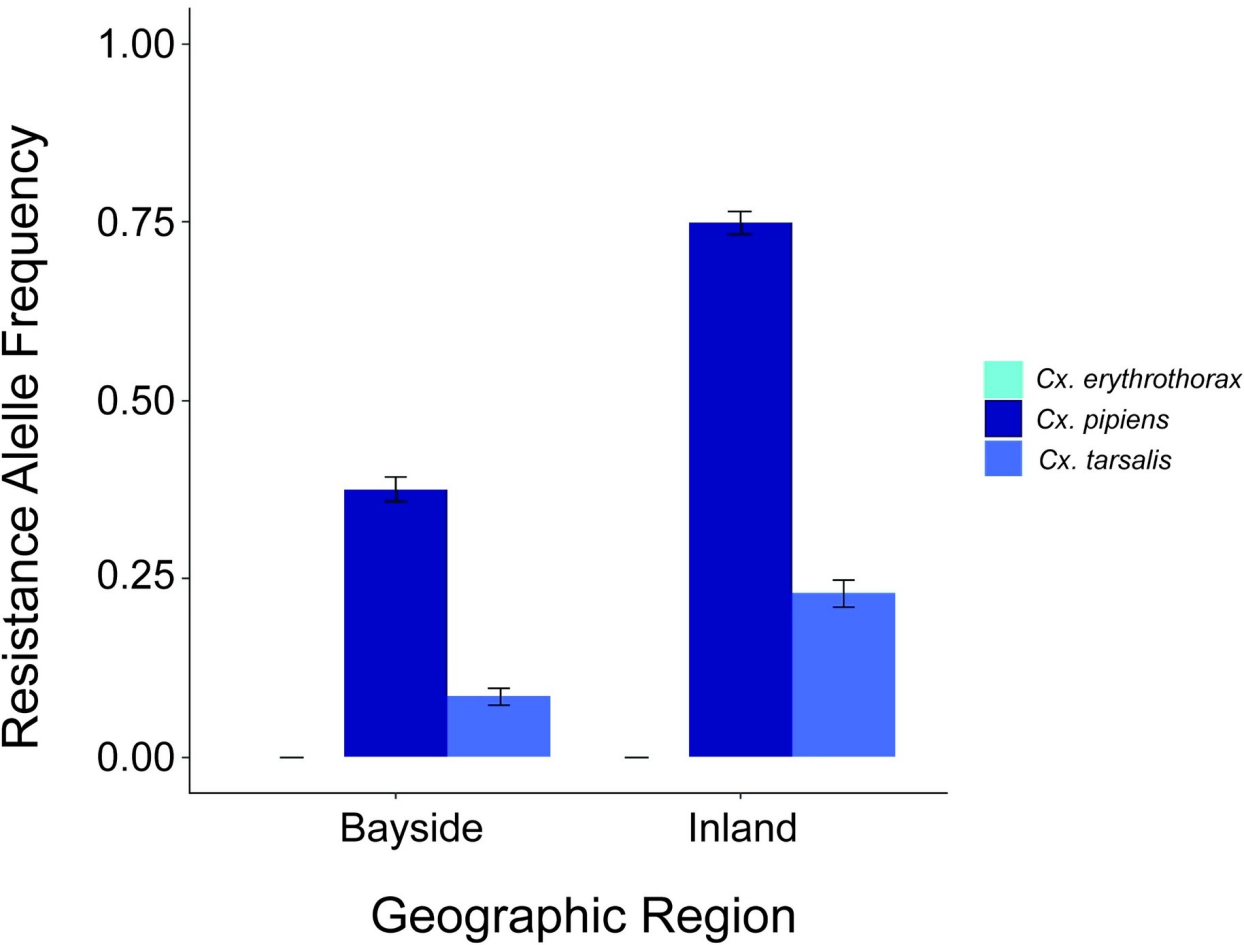
Resistant allele frequency (F_(FF,LF)_) of the L1014F *kdr* mutation by species and region. Bright blue, dark blue and medium blue bars represent F_(FF,LF)_ for *Cx*. *erythrothorax*, *Cx*. *pipiens* and *Cx*. *tarsalis*, respectively. The F_(FF,LF)_ for bayside *Cx*. *pipiens* and *Cx*. *tarsalis* was 0.375 ± 0.018 and 0.0840 ± 0.012, respectively. The F_(FF,LF)_ for inland *Cx pipiens* and *Cx*. *tarsalis* was 0.749 ± 0.016 and 0.230 ± 0.016, respectively.

The *Culex* RT*kdr* assay was used to assess the geographic distribution of the L1014F mutation in Alameda County (Fig 6A and 6B). Among the *Culex spp*. individuals that were tested, 26.2% were homozygous FF-1014, 20.6% were heterozygous LF-1014, and 53.3% were homozygous LL-1014 (N = 1383 mosquitoes). Ordinal logistic regression was used to determine associations between genotype, mosquito species, region of collection and land use type (agricultural, industrial, urban and wildlife). There was a greater proportion of agricultural sites within the inland region relative to bayside (33% and 4%, respectively; Fig 6C). The bayside region had a greater proportion of wildlife sites compared to the inland region (35% and 10%, respectively; Fig 6C). Because the L1014F *kdr* mutation was not found in *Cx*. *erythrothorax*, ordinal logistic regression models were fit only to *Cx*. *pipiens* and *Cx*. *tarsalis* data. The overall resistant allele frequency (F_(FF,LF)_) was highest among *Cx*. *pipiens* (0.57), low for *Cx*. *tarsalis* (0.15) and not present for *Cx*. *erythrothorax* (0.00; Table 5). *Culex pipiens* had 8.99 times greater odds of being LF-1014 or FF-1014 compared to *Cx*. *tarsalis* (Table 5, 95%CI: 6.96–11.69). Adjusting for region and land use type increased the association between resistance and *Cx*. *pipiens* (Table 5; OR: 11.01 (8.36–14.63)) suggesting an association present between *Cx*. *pipiens* mosquitoes and higher levels of resistance. The inland region had a higher F_(FF,LF)_ compared to the bayside region for both *Cx pipiens* and *Cx*. *tarsalis* (Fig 7). *Culex erythrothorax* was not present in the inland region during the study period and all bayside *Cx*. *erythrothorax* were homozygous LL-1014.

**Table 5:** Genotypes detected, F_(FF,LF)_, unadjusted and adjusted odds ratios among species, geographic region and land use type. Homozygous LL-1014 (LL), heterozygous LF-1014 (LF), homozygous FF-1014 (FF).

Variable		Genotype				Odds Ratio, OR (95% CI)	
	N	LL	LF	FF	F_(FF,LF)_	Unadjusted	Adjusted
**Species**							
*Cx*. *erythrothorax*	126	126	0	0	0	NA	NA
*Cx*. *tarsalis*	507	401	57	49	0.15	Ref	Ref
*Cx*. *pipiens*	744	208	226	310	0.57	8.99 (6.98–11.69, p < 0.001)	11.01 (8.36–14.63, p < 0.001)
**Region**							
Bayside	744	519	136	89	0.21	Ref	Ref
Inland	633	216	147	270	0.54	3.92 (3.15–4.89, p < 0.001)	4.89 (3.79–6.33, p < 0.001)
**Land use type**							
Wildlife	484	296	94	94	0.29	Ref	Ref
Urban	303	123	80	100	0.46	1.95 (1.47–2.58, p < 0.001)	0.96 (0.70–1.32, p = 0.817)
Industrial	449	251	83	115	0.35	1.13 (0.87–1.46, p = 0.367)	0.77 (0.57–1.03, p = 0.080)
Agriculture	141	65	26	50	0.45	1.74 (1.21–2.53, p = 0.003)	0.89 (0.58–1.37, p = 0.604)

High resistant allelic frequencies were found previously in *Cx*. *pipiens* complex mosquitoes [11, 53]. *Culex erythrothorax* reproduce in heavily vegetated regions of shallow ponds and can be highly abundant in marsh habitats [54, 55]. While *Cx*. *erythrothorax* were typically found in bayside wetlands, *Cx*. *pipiens* and *Cx*. *tarsalis* were both present inland, yet the L1014F mutation associated with pyrethroid resistance was more common for *Cx*. *pipiens*. Waterways near agricultural fields can contain high levels of pyrethroids that contaminate the water and sediment. Similar levels of pyrethroid contamination have been observed in the urban creeks and storm drain outfalls of California that likely originated from residential turf and structural pest control efforts [27, 56]. Because immature *Cx*. *pipiens* and *Cx*. *tarsalis* can develop in urban waterways, persistent exposure to pyrethroids in the water and sediment may have exerted a selective pressure to establish the LF-1014 and FF-1014 genotypes that were observed.

Mosquitoes from inland regions of Alameda County had elevated odds of having the L1014F SNP that is associated with pyrethroid resistance (Table 5; OR: 3.92 (3.15–4.89, p < 0.001)). Adjusting for species and land use type increased the association between genotypes that are linked to pyrethroid resistance (LF-1014 and FF-1014) and mosquitoes that were collected from inland sites (OR: 4.89 (3.79–6.33, p < 0.001)), suggesting an association between inland mosquitoes and potential for higher levels of pyrethroid resistance. Unadjusted odds ratios showed evidence of an association between resistant genotypes (LF-1014 and FF-1014) and urban or agriculture land use types (Table 5; OR_urban_: 1.95 (1.47–2.58, p < 0.001), OR_agriculture_: 1.74 (1.21–2.53, p = 0.003)), but these associations were not significant when adjusting for species and region (Table 5; OR_urban_: 0.96 (0.70–1.32, p = 0.817), OR_agriculture_: 0.89 (0.58–1.37, p = 0.604)). Pesticides are used in California (USA) predominantly to control agricultural and structural pests; public health use of pesticides accounts for less than 1% of the total [57]. The California Pesticide Information Portal (CPIP) shows that the top uses of pyrethroids in Alameda County were for structural pest control, wine grapes, almonds, and brussels sprouts [58]. Although the CPIP did not specify townships where insecticides are applied for structural pest control, CPIP and Pesticide Use Report (PUR) pointed to locations within the inland region of Alameda County with insecticide applications for agriculture. Agriculture is more widely practiced within the inland region of Alameda County relative to the bayside region of Alameda County. Studies of *Anopheles gambiae*, the malaria mosquito, suggest that insecticides from agriculture likely contribute to insecticide resistance [28, 59, 60]. A similar pattern of pyrethroid use in agriculture cooccurring with pyrethroid resistance was observed in *Cx*. *pipiens* and *Cx*. *tarsalis*, two important vectors of WNV in North America.

## Conclusion

We developed a high throughput RT-qPCR assay that was accurate in six *Culex spp*. for detecting the L1014 *kdr* mutation. DNA-based assays that detect the L1014 SNP in *Culex spp*. are available [16–18], some of which rely upon Sanger sequencing a PCR product. A genomic DNA-based quantitative PCR assay was previously reported that identified the L1014F SNP in the *kdr* loci of *Culex pipiens* [17]. While it is of use for that species, unlike the *Culex* RT*kdr* assay reported herein, it is unlikely able to detect the mutation in other *Culex spp*. as those primers span an intron region, which is not conserved across species. Because the primers and probes of the *Culex* RT*kdr* assay bind to a conserved region of an exon, it was effective for six *Culex spp*. (Table 4). The previously reported DNA-based quantitative PCR [17] and *Culex* RT*kdr* assays had similar accuracy. An advantage of the *Culex* RT*kdr* assay over others is that RNA rather than DNA is used. This allows vector control workers to utilize the same nucleic acid purification and quantitative RT-PCR reagents that they routinely use to assess arbovirus prevalence in mosquitoes, thereby conserving public funds. Utilizing the *Culex* RT*kdr* assay rather than individual DNA-based assays for each species simplifies the workflow of vector control labs and saves agencies from needing to develop additional standard operation procedures and worker proficiency assessments.

Like all PCR-based assays, the *Culex* RT*kdr* assay is not without limitations. The assay did not detect the SF-1014 SNP in the *kdr* locus (*i*.*e*., L1014S) that was discovered by sequencing the RT*kdr* assay PCR product from the Conaway strain (Fig 5) and previously in *Cx*. *pipiens* complex mosquitoes [16]. However, the L1014F allele is the most common SNP in the *Vgsc* to be associated with resistance to pyrethroid insecticides [5]. The *Culex* RT*kdr* assay also does not account for other pyrethroid resistance mechanisms such as overexpression or mutation of CYP9M10. Overexpression of CYP9M10 allows for increased detoxification of pyrethroids by this cytochrome P450 monooxygenase [5, 47]. The *Culex* RT*kdr* assay was extensively validated for only *Cx*. *pipiens*, Cx. *tarsalis* and *Cx*. *erythrothorax* mosquitoes because we had a limited number of other *Culex* species available for the study. However, preliminary results suggest the assay performs for *Cx*. *quinquefasciatus*, *Cx*. *apicalis* and *Cx*. *stigmatosoma*. Lastly, the *Culex* RT*kdr* assay performs well using Northern California mosquitoes, but regional genetic diversity may prevent the assay from detecting the L1014F mutation in *Culex* species worldwide. Promisingly, the variations within the non-coding region should not limit the assay as the *Culex* RT*kdr* exploits reverse transcriptase that converts RNA to cDNA, thus non-coding regions are excluded from the template. More research is needed to determine whether this assay could be applied to mosquitoes collected outside of California.

Despite public health pesticide applications accounting for <1% of statewide pesticide use from 1993–2007, and with Alameda County Mosquito Abatement District having applied less than 300 milliliters of adulticide in the decade covering 2010 to 2020, pyrethroid resistance remains a concern [57]. Commercial use of insecticides for both structural and agricultural pest control may contribute to higher pyrethroid resistance in mosquitoes from the inland region. In countries that ceased pyrethroid applications by vector control agencies, resistance remained high, likely due to household insecticides that contain pyrethroids [61, 62].

It may be possible to employ the approach used to develop the Culex RT*kdr* assay for other mosquito species. The V*gsc* sequences of *Aedes aegpti* Linnaeus and *Aedes albopictus* Skuse have a high percent nucleotide identity around the V1016G *kdr* mutation, suggesting the development of an *Aedes* RT*kdr* assay may be possible [52]. Application of pyrethroids to a resistant population can potentially drive heterozygous populations (LF-1014) to the homozygous resistant genotype (FF-1014), thereby increasing the frequency of FF-1014 as ineffective insecticides are released into the environment. Prior to the development of this *Culex* RT*kdr*, there was no quantitative PCR assay to detect the L1014F mutation in *Cx*. *tarsalis*. The development of our *Culex* RT*kdr* assay satiates the need for a simple and reliable PCR-based assay for detecting a marker of pyrethroid resistance in *Cx*. *tarsalis*. We hope the assay will improve testing for pyrethroid resistance among *Culex* species.

## Supporting information

S1 File Code used in R software to assess geographic distribution of LL-1014, LF-1014, and FF-1014 in *Cx*. *pipiens* and *Cx*. *tarsalis* that were collected in Alameda County using EVS traps. (DOCX)

S1 Fig *Culex* RT*kdr* assay amplification plots for **(A)** *Cx*. *quinquefasciatus* strain CqWV-1 (N = 10, each was LL-1014), **(B)** *Cx*. *quinquefasciatus* strain CqWV-2 (N = 10, each was FF-1014), **(C)** *Cx*. *stigmatosoma* (N = 3, each was LF-1014), and **(D)** *Cx*. *apicalis* (N = 3, two were FF-1014, one was LF-1014).
(TIF)
