# Supplementary material for: Quantitative reverse transcription PCR assay to detect a genetic marker of pyrethroid resistance in Culex mosquitoes
Source: PLoS One. 2022 Aug 8;17(8):e0252498. doi: 10.1371/journal.pone.0252498 (PMC9359573; doi:10.1371/journal.pone.0252498)
Supplement: S1 File — (DOCX) [file pone.0252498.s001.docx]

## Figure S1

###

**Supplementary methods and data for statistical analysis of *kdr* genotype by species, region, and land use type in Table 5**

### R Software Code

Supplementary 1

# Loading in and cleaning Resistance data
 resist_data <- readxl::read_excel("~/Box/Fall 2021/Mosquitoes/Data Files/Resistance by species and region.xlsx",
 sheet = "All") %>%
 mutate(`Area Type` = case_when(`Area Type` %in% c("Agriculture", "Agricutlure") ~ "Agriculture", # Fix some typos in land use type
 `Area Type` %in% c("Idustrial", "Industrial") ~ "Industrial",
 `Area Type` %in% c("Uban", "Urban") ~ "Urban",
 `Area Type` %in% c("Wildlife", "Wildliffe") ~ "Wildlife"),
 # Add variable for number of alleles
 alleles = case_when(`Mutant Type` == 1 ~ 2,
 Heterozygous == 1 ~ 1,
 `Wild Type` == 1 ~ 0),
 # Add variable for resistance type
 resist_type = case_when(`Mutant Type` == 1 ~ "Resistant",
 Heterozygous == 1 ~ "Heterozygous",
 `Wild Type` == 1 ~ "Susceptible"),
 # Region condensed to inland vs Bayside
 region2 = if_else(`County Region` == "East", "Inland", "Bayside"),
 # Convert to factor with appropriate levels
 resist_type = factor(resist_type, levels = c("Susceptible", "Heterozygous", "Resistant")),
 # Create variable for resistance phenotype assuming heterozygosity confers resistance
 resist_phenotype = if_else(resist_type == "Susceptible", "Susceptible", "Resistant"))

**Methods and Equations**

Analyses of resistant and susceptible alleles were assessed by mosquito species and by geographic region (inland and coastal) within Alameda County. The frequency of alleles that are associated with pyrethroid resistance (F_(FF,LF)_) in each population was estimated as:

$$F_{(FF,LF)}=\frac{2N_{FF}+N_{LF}}{2N}$$

where $N_{FF}$ is the number of homozygous resistant mosquitoes, $N_{LF}$ the number of heterozygous resistant, and $N$ the mosquito population size.

Associations between genotype, $Y$, and mosquito species, region of collection, and land use surrounding the collection site were estimated from an ordinal logistic regression model with ordered outcome categories $j\in\{LL,LF,FF\}$:

$$logit(P(Y\leq j))=\beta_{0_{j}}-\beta_{1}Species-\beta_{2}Region-\beta_{3}LandUse$$

The model was fit using the polr function from the MASS package in R version 3.5.0 and used to estimate unadjusted and adjusted odds ratios for each variable. Adjusted odds ratios are derived from a saturated model that includes all covariates at once whereas unadjusted odds ratios are derived from models with only the covariate of interest included.

**Data Output of R Code for Ordinal Regression Models**

*Culex erythrothorax* was excluded from models because no resistant alleles (LF-1014 or FF-1014) were observed in this study for that species.

# Ordinal Regression: Species Model (unadjusted)
resistance_olr_species <- polr(resist_type ~ Species,
 data = resist_data %>%
 filter(Species %in% c("Culex pipiens", "Culex tarsalis")) %>%
 mutate(Species = factor(Species, levels = c("Culex tarsalis", "Culex pipiens"))),
 Hess = T)

OR_pip <- exp(coef(resistance_olr_species))
confint_pip <- exp(confint(resistance_olr_species))

OR2.5_pip <- confint_pip[1]
OR97.5_pip <- confint_pip[2]


pvals_pip_init <- coef(summary(resistance_olr_species))
pvals_pip <- pnorm(abs(pvals_pip_init[, "t value"]), lower.tail = FALSE) * 2

(Pip_stats <- cbind(pvals_pip_init, "p value" = pvals_pip))

## Value Std. Error t value p value
## SpeciesCulex pipiens 2.195807 0.1321458 16.61655 5.289300e-62
## Susceptible|Heterozygous 1.302001 0.1089723 11.94800 6.650375e-33
## Heterozygous|Resistant 2.496551 0.1239365 20.14379 3.050816e-90

pval_pip <- pvals_pip[1]

# Ordinal Regression: Region Model (unadjusted)

resistance_olr_region <- polr(resist_type ~ region2,
 data = resist_data %>%
 filter(Species %in% c("Culex pipiens", "Culex tarsalis")),
 Hess = T)

OR_inland <- exp(coef(resistance_olr_region))
confint_inland <- exp(confint(resistance_olr_region))

OR2.5_inland <- confint_inland[1]
OR97.5_inland <- confint_inland[2]

pvals_inland_init <- coef(summary(resistance_olr_region))
pvals_inland <- pnorm(abs(pvals_inland_init[, "t value"]), lower.tail = FALSE) * 2

(Region_stats <- cbind(pvals_inland_init, "p value" = pvals_inland))

## Value Std. Error t value p value
## region2Inland 1.3650987 0.11237577 12.147625 5.905636e-34
## Susceptible|Heterozygous 0.6082443 0.08175281 7.440042 1.006530e-13
## Heterozygous|Resistant 1.6789999 0.09363538 17.931254 6.724535e-72

pval_inland <- pvals_inland[1]

# Ordinal Regression: Land Use Model (unadjusted)

resistance_olr_area <- polr(resist_type ~ `Area Type`,
 data = resist_data %>%
 filter(Species %in% c("Culex pipiens", "Culex tarsalis")) %>%
 mutate(`Area Type` = factor(`Area Type`,
 levels = c("Wildlife", "Urban", "Industrial", "Agriculture"))),
 Hess = T)

OR_urban <- exp(coef(resistance_olr_area)[which(grepl("Urban", names(coef(resistance_olr_area))))])
OR_industrial <- exp(coef(resistance_olr_area)[which(grepl("Industrial", names(coef(resistance_olr_area))))])
OR_agriculture <- exp(coef(resistance_olr_area)[which(grepl("Agriculture", names(coef(resistance_olr_area))))])

confint_area <- exp(confint(resistance_olr_area))

OR2.5_urban <- confint_area[1,1]
OR97.5_urban <- confint_area[1,2]

OR2.5_industrial <- confint_area[2,1]
OR97.5_industrial <- confint_area[2,2]

OR2.5_agriculture <- confint_area[3,1]
OR97.5_agriculture <- confint_area[3,2]

pvals_area_init <- coef(summary(resistance_olr_area))
pvals_area <- pnorm(abs(pvals_area_init[, "t value"]), lower.tail = FALSE) * 2

(Land_Use_stats <- cbind(pvals_area_init, "p value" = pvals_area))

## Value Std. Error t value p value
## `Area Type`Urban 0.6665758 0.14376233 4.636652 3.540986e-06
## `Area Type`Industrial 0.1196791 0.13274703 0.901558 3.672917e-01
## `Area Type`Agriculture 0.5566348 0.18847919 2.953296 3.144004e-03
## Susceptible|Heterozygous 0.1979145 0.09550506 2.072293 3.823813e-02
## Heterozygous|Resistant 1.1787545 0.10129328 11.637045 2.671095e-31

pval_urban <- pvals_area[1]
pval_industrial <- pvals_area[2]
pval_agriculture <- pvals_area[3]

# Ordinal Regression: Saturated Model (Adjusted)
resistance_olr_all <- polr(resist_type ~ `Area Type` + Species + region2,
 data = resist_data %>%
 filter(Species %in% c("Culex pipiens", "Culex tarsalis")) %>%
 mutate(Species = factor(Species,
 levels = c("Culex tarsalis", "Culex pipiens")),
 `Area Type` = factor(`Area Type`,
 levels = c("Wildlife", "Urban", "Industrial", "Agriculture"))),
 Hess = T)

OR_pip_adj <- exp(coef(resistance_olr_all)[which(grepl("pipiens", names(coef(resistance_olr_all))))])

OR_inland_adj <- exp(coef(resistance_olr_all)[which(grepl("Inland", names(coef(resistance_olr_all))))])

OR_urban_adj <- exp(coef(resistance_olr_all)[which(grepl("Urban", names(coef(resistance_olr_all))))])
OR_industrial_adj <- exp(coef(resistance_olr_all)[which(grepl("Industrial", names(coef(resistance_olr_all))))])
OR_agriculture_adj <- exp(coef(resistance_olr_all)[which(grepl("Agriculture", names(coef(resistance_olr_all))))])

confint_all <- exp(confint(resistance_olr_all))

## Waiting for profiling to be done...

pvals_init <- coef(summary(resistance_olr_all))
pvals <- pnorm(abs(pvals_init[, "t value"]), lower.tail = FALSE) * 2

(Adjusted_stats <- cbind(pvals_init, "p value" = pvals))

## Value Std. Error t value p value
## `Area Type`Urban -0.03765631 0.1625636 -0.2316404 8.168173e-01
## `Area Type`Industrial -0.26296539 0.1500753 -1.7522229 7.973549e-02
## `Area Type`Agriculture -0.11284360 0.2175008 -0.5188192 6.038868e-01
## SpeciesCulex pipiens 2.39910671 0.1428558 16.7939001 2.704721e-63
## region2Inland 1.58720482 0.1308764 12.1275052 7.551509e-34
## Susceptible|Heterozygous 2.10220719 0.1564983 13.4327805 3.885442e-41
## Heterozygous|Resistant 3.46790107 0.1777825 19.5064220 9.682665e-85

confint_all <- cbind(confint_all, pvals)

## Warning in cbind(confint_all, pvals): number of rows of result is not a multiple
## of vector length (arg 2)

OR2.5_urban_adj <- confint_all[1,1]
OR97.5_urban_adj <- confint_all[1,2]
pval_urban_adj <- confint_all[1,3]

OR2.5_industrial_adj <- confint_all[2,1]
OR97.5_industrial_adj <- confint_all[2,2]
pval_industrial_adj <- confint_all[2,3]

OR2.5_agriculture_adj <- confint_all[3,1]
OR97.5_agriculture_adj<- confint_all[3,2]
pval_agriculture_adj<- confint_all[3,3]

OR2.5_pip_adj <- confint_all[4,1]
OR97.5_pip_adj <- confint_all[4,2]
pval_pip_adj <- confint_all[4,3]

OR2.5_inland_adj <- confint_all[5,1]
OR97.5_inland_adj <- confint_all[5,2]
pval_inland_adj <- confint_all[5,3]

#Summarize species data for table

resist_data_species <- resist_data %>%
 filter(Species %in% c("Culex erythrothorax", "Culex pipiens", "Culex tarsalis")) %>%
 mutate(Species = factor(Species, levels = c("Culex erythrothorax", "Culex tarsalis", "Culex pipiens"))) %>%
 group_by(Species) %>%
 summarise(N = sum(!is.na(alleles)),
 n_alleles = N*2,
 LL = sum(alleles == 0, na.rm = T),
 LF = sum(alleles == 1, na.rm = T),
 FF = sum(alleles == 2, na.rm = T),
 n_R_alleles = sum(alleles[!is.na(alleles)]),
 n_S_alleles = n_alleles - n_R_alleles,
 `$F_R$` = round(n_R_alleles/n_alleles, 2),
 F_R_se = sqrt((`$F_R$`*(1-`$F_R$`))/n_alleles)) %>%
 mutate(Variable = case_when(Species == "Culex erythrothorax" ~ "Cx. erythrothorax",
 Species == "Culex tarsalis" ~ "Cx. tarsalis",
 Species == "Culex pipiens" ~ "Cx. pipiens"),
 OR = c(NA_real_, "Ref", round(OR_pip, 2)),
 OR2.5 = c(NA_real_, "Ref", round(OR2.5_pip, 2)),
 OR97.5 = c(NA_real_, "Ref", round(OR97.5_pip, 2)),
 pval = pround(pval_pip),
 Unadjusted = case_when(is.na(OR) ~ NA_character_,
 OR == "Ref" ~ "Ref",
 TRUE ~ paste0(OR, " (", OR2.5, " - ", OR97.5,
 ", p ", pval, ")")),
 OR_adj = c(NA_real_, "Ref", round(OR_pip_adj, 2)),
 OR2.5_adj = c(NA_real_, "Ref", round(OR2.5_pip_adj, 2)),
 OR97.5_adj = c(NA_real_, "Ref", round(OR97.5_pip_adj, 2)),
 pval_adj = pround(pval_pip_adj),
 Adjusted = case_when(is.na(OR_adj) ~ NA_character_,
 OR_adj == "Ref" ~ "Ref",
 TRUE ~ paste0(OR_adj, " (", OR2.5_adj, " - ", OR97.5_adj,
 ", p ", pval_adj, ")"))) %>%
 select(Variable, N, LL, LF, FF, `$F_R$`, Unadjusted, Adjusted)

#Summarize region data for table

resist_data_region <- resist_data %>%
 filter(Species %in% c("Culex erythrothorax", "Culex pipiens", "Culex tarsalis")) %>%
 group_by(region2) %>%
 summarise(N = sum(!is.na(alleles)),
 n_alleles = N*2,
 LL = sum(alleles == 0, na.rm = T),
 LF = sum(alleles == 1, na.rm = T),
 FF = sum(alleles == 2, na.rm = T),
 n_R_alleles = sum(alleles[!is.na(alleles)]),
 n_S_alleles = n_alleles - n_R_alleles,
 `$F_R$` = round(n_R_alleles/n_alleles, 2),
 F_R_se = sqrt((`$F_R$`*(1-`$F_R$`))/n_alleles)) %>%
 mutate(Variable = region2,
 OR = c("Ref", round(OR_inland, 2)),
 OR2.5 = c("Ref", round(OR2.5_inland, 2)),
 OR97.5 = c("Ref", round(OR97.5_inland, 2)),
 pval = pround(pval_pip_adj),
 Unadjusted = case_when(OR == "Ref" ~ "Ref",
 TRUE ~ paste0(OR, " (", OR2.5, " - ", OR97.5,
 ", p ", pval, ")")),
 OR_adj = c("Ref", round(OR_inland_adj, 2)),
 OR2.5_adj = c("Ref", round(OR2.5_inland_adj, 2)),
 OR97.5_adj = c("Ref", round(OR97.5_inland_adj, 2)),
 pval_adj = pround(pval_inland_adj),
 Adjusted = case_when(OR_adj == "Ref" ~ "Ref",
 TRUE ~ paste0(OR_adj, " (", OR2.5_adj, " - ", OR97.5_adj,
 ", p ", pval_adj, ")"))) %>%
 select(Variable, N, LL, LF, FF, `$F_R$`, Unadjusted, Adjusted)

#Summarize region data for table
resist_data_area <- resist_data %>%
 mutate(Area = factor(`Area Type`, levels = c("Wildlife", "Urban", "Industrial", "Agriculture"))) %>%
 group_by(Area) %>%
 summarise(N = sum(!is.na(alleles)),
 n_alleles = N*2,
 LL = sum(alleles == 0, na.rm = T),
 LF = sum(alleles == 1, na.rm = T),
 FF = sum(alleles == 2, na.rm = T),
 n_R_alleles = sum(alleles[!is.na(alleles)]),
 n_S_alleles = n_alleles - n_R_alleles,
 `$F_R$` = round(n_R_alleles/n_alleles, 2),
 F_R_se = sqrt((`$F_R$`*(1-`$F_R$`))/n_alleles)) %>%
 filter(!is.na(Area)) %>%
 mutate(Variable = Area,
 OR = c("Ref", round(c(OR_urban, OR_industrial, OR_agriculture), 2)),
 OR2.5 = c("Ref", round(c(OR2.5_urban, OR2.5_industrial, OR2.5_agriculture), 2)),
 OR97.5 = c("Ref", round(c(OR97.5_urban, OR97.5_industrial, OR97.5_agriculture), 2)),
 pval = c("Ref", pround(pval_urban), pround(pval_industrial), pround(pval_agriculture)),
 Unadjusted = case_when(OR == "Ref" ~ "Ref",
 TRUE ~ paste0(OR, " (", OR2.5, " - ", OR97.5,
 ", p ", pval, ")")),
 OR_adj = c("Ref", round(c(OR_urban_adj, OR_industrial_adj, OR_agriculture_adj), 2)),
 OR2.5_adj = c("Ref", round(c(OR2.5_urban_adj, OR2.5_industrial_adj, OR2.5_agriculture_adj), 2)),
 OR97.5_adj = c("Ref", round(c(OR97.5_urban_adj, OR97.5_industrial_adj, OR97.5_agriculture_adj), 2)),
 pval_adj = c("Ref", pround(pval_urban_adj), pround(pval_industrial_adj), pround(pval_agriculture_adj)),
 Adjusted = case_when(OR_adj == "Ref" ~ "Ref",
 TRUE ~ paste0(OR_adj, " (", OR2.5_adj, " - ", OR97.5_adj,
 ", p ", pval_adj, ")"))) %>%
 select(Variable, N, LL, LF, FF, `$F_R$`, Unadjusted, Adjusted)

# Pretty pub table
rbind(resist_data_species,
 resist_data_region,
 resist_data_area) %>%
 mutate_all(linebreak) %>%
 knitr::kable("latex", booktabs = T, escape = F,
 caption = "Summary of statistical analyses") %>%
 pack_rows("Species", 1, 3) %>%
 pack_rows("Region", 4, 5) %>%
 pack_rows("Area Type", 6, 9) %>%
 add_header_above(c(" ", " ", "Genotype"=3, " ","Summary Statistics, OR (95% CI, p-value) "=2)) %>%
 kable_styling(latex_options = "striped")

resist_data_sum2 <- resist_data %>%
 filter(Species %in% c("Culex erythrothorax",
 "Culex pipiens",
 "Culex tarsalis")) %>%
 group_by(region2, Species) %>%
 summarise(N = sum(!is.na(alleles)),
 n_alleles = N*2,
 n_susc = sum(alleles == 0, na.rm = T),
 n_hetero = sum(alleles == 1, na.rm = T),
 n_resist = sum(alleles == 2, na.rm = T),
 n_R_alleles = sum(alleles[!is.na(alleles)]),
 n_S_alleles = n_alleles - n_R_alleles,
 allele_freq = n_R_alleles/n_alleles,
 allele_freq_se = sqrt((allele_freq*(1-allele_freq))/n_alleles))

## `summarise()` has grouped output by 'region2'. You can override using the
## `.groups` argument.

allele_freq_species_region2 <- resist_data_sum2 %>%
 filter(Species %in% c("Culex erythrothorax",
 "Culex pipiens",
 "Culex tarsalis")) %>%
 ggplot(aes(x = region2, y = allele_freq, fill = Species)) +
 geom_bar(stat = "identity", position = position_dodge()) +
 geom_errorbar(aes(ymin = allele_freq - allele_freq_se,
 ymax = allele_freq + allele_freq_se),
 width = 0.2,
 position = position_dodge(0.9)) +
 theme_classic() +
 theme(axis.text = element_text(size = 10),
 axis.title = element_text(size = 12)) +
 ylim(c(0,1)) +
 labs(x = "Region",
 y = "Resistance Allele Frequency",
 title = "Resistance allele frequency by region and species") +
 scale_fill_manual(values = c("grey90", "grey50", "grey10"))
