# Supplementary figures and images for: Quantitative reverse transcription PCR assay to detect a genetic marker of pyrethroid resistance in Culex mosquitoes
Source: PLoS One. 2022 Aug 8;17(8):e0252498. doi: 10.1371/journal.pone.0252498 (PMC9359573; doi:10.1371/journal.pone.0252498)

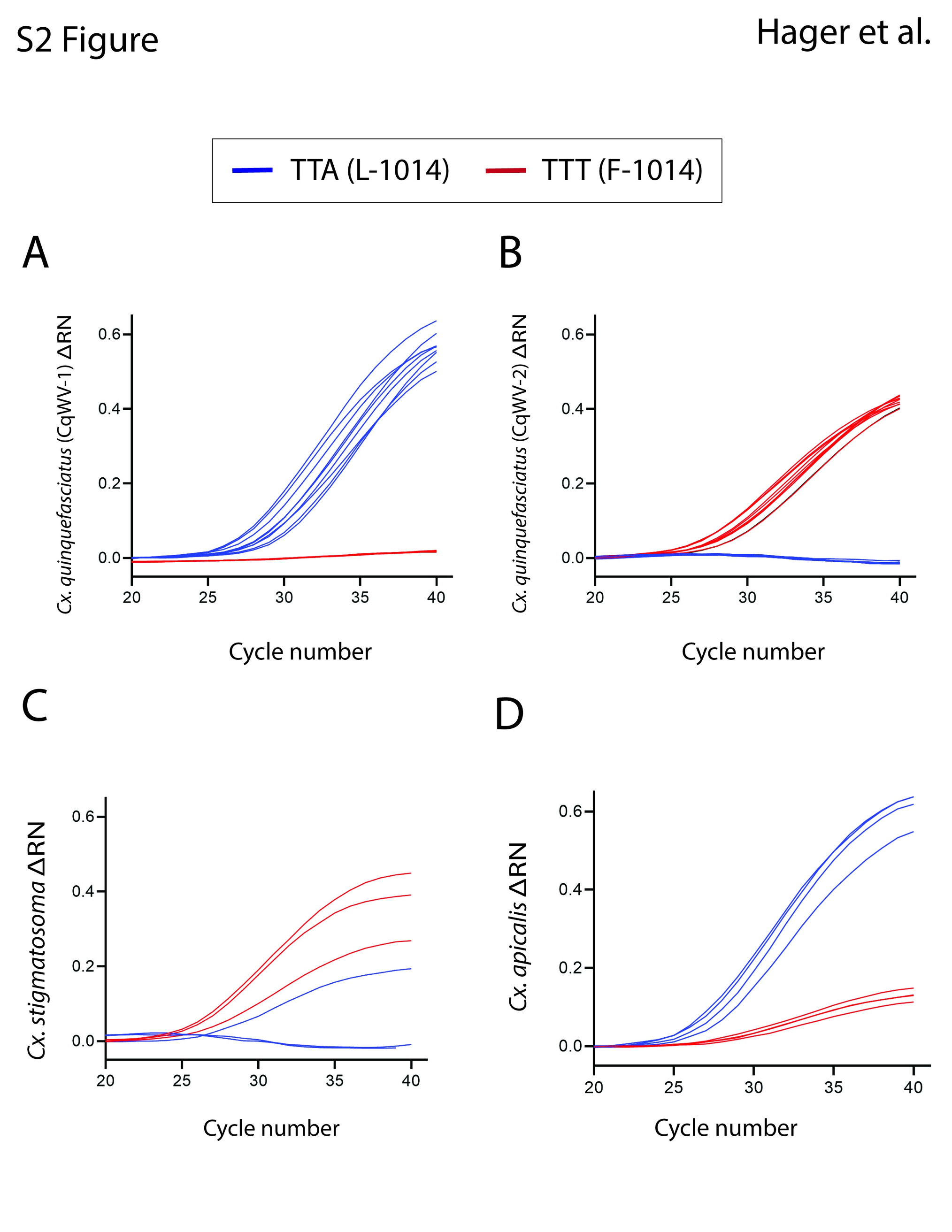

Supplement: S1 Fig — Culex RTkdr assay amplification plots for (A) Cx. quinquefasciatus strain CqWV-1 (N = 10, each was LL-1014), (B) Cx. quinquefasciatus strain CqWV-2 (N = 10, each was FF-1014), (C) Cx. stigmatosoma (N = 3, each was LF-1014), and (D) Cx. apicalis (N = 3, two were FF-1014, one was LF-1014). (TIF) [file pone.0252498.s002.tif]
